# Insufficient evidence to conclude that confusion cannot explain cooperative behavior

**DOI:** 10.1073/pnas.2412216121

**Published:** 2024-08-16

**Authors:** Toke R. Fosgaard, Erik Wengström

**Affiliations:** ^a^Department of Technology, Management, and Economics, Technical University of Denmark, Kgs Lyngby 358 2800, Denmark; ^b^Department of Economics, Lund University, Lund 223 63, Sweden

We read with interest the paper by Wang et al. (2024) on confusion in public goods games (1). Their conclusion—that confusion is not a significant factor in explaining cooperation— challenges much of the existing literature (2–5). However, we believe that their study overlooks crucial aspects of confusion measurement and lacks critical design elements.

Wang et al. suggest that the use of 10 control questions with feedback explains the low levels of confusion they find. They argue that the confusion observed in previous studies is due to insufficient instruction. However, this conclusion is drawn without comparing their design to a baseline treatment similar to earlier studies with fewer control questions and less feedback. Such a comparison is essential to substantiate their claim. Without it, the interpretation of their findings remains speculative.

Moreover, Wang et al. also test confusion by comparing public good contributions in interaction between human group members with interaction with computerized group members—an often-used method (2). Wang et al. observe substantial cooperation even in their computer treatment, which seems to contradict their assertion that confusion is not an issue. This finding itself raises questions about the validity of their claim.

Furthermore, their study does not engage with earlier literature using similar but more comprehensive multiitem ways of measuring confusion. For example, Fosgaard et al. (5) test participants’ understanding of both sides of the social dilemma through an incentivized task. Their six-item task captures the understanding of the payoff-maximizing strategy as well as the understanding of the socially optimal strategy. Such tests are better suited for ascertaining whether participants grasp the social dilemma of engaging in public good contribution and, if not, in which direction participants’ errors occur.

Results from Fosgaard et al. (5), shown in Fig. 1, highlight that substantial confusion is detected with the more comprehensive measures and that confusion exists on both sides of the social dilemma. Moreover, varying the way the public good game is framed—Give vs. Take framing—substantially affects the measured degree of confusion. Ferraro and Vossler (3) also indicate this finding of treatment-dependent confusion, reporting that labeling contributions as donations rather than investments leads to lower contribution levels when other group members are computers. Given this, making bold conclusions about the importance of confusion based on one treatment without proper baselines, as Wang et al. (1), appears premature.

**Fig. 1.**
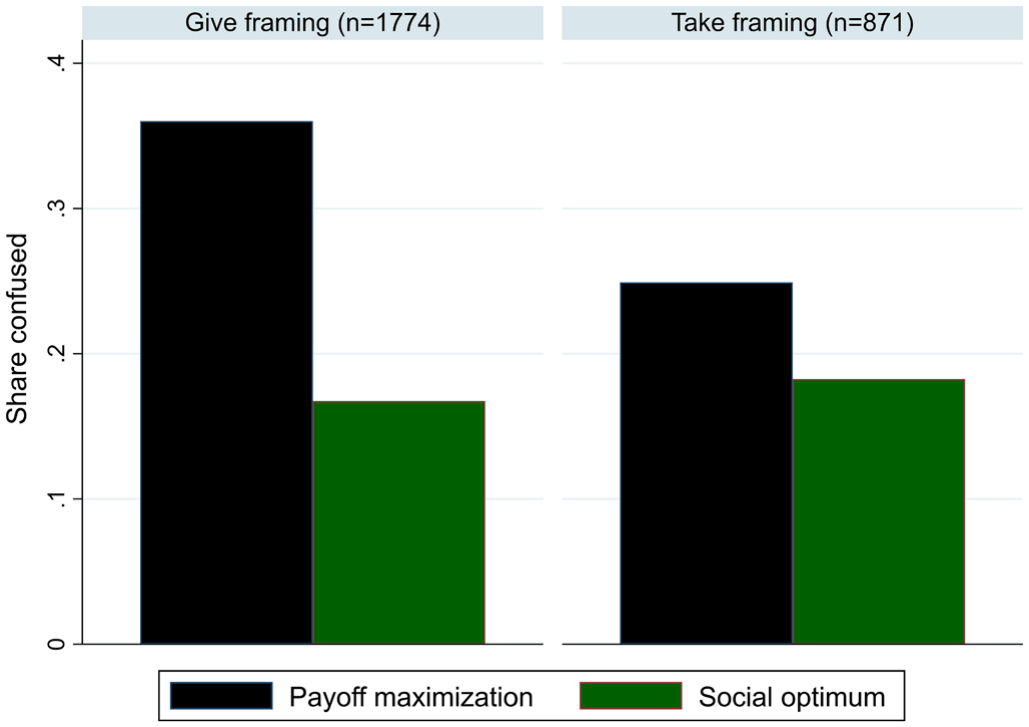
Measures capturing confusion about the payoff-maximizing strategy and social optimal strategy in the public good game across the traditionally used give framing and the alternative take framing.

In conclusion, while Wang et al. provide intriguing evidence of confusion in public goods games, their findings risk underestimating its role in shaping cooperation and responses to experimental treatments.

## References

[1] G. Wang , Confusion cannot explain cooperative behavior in public goods games. Proc. Natl. Acad. Sci. U.S.A. **121**, e2310109121 (2024), 10.1073/pnas.2310109121. 38412126 PMC10927562

[2] D. Houser, R. Kurzban, Revisiting kindness and confusion in public goods experiments. Am. Econ. Rev. **92**, 1062–1069 (2002).

[3] P. J. Ferraro, C. A. Vossler, The source and significance of confusion in public goods experiments. B.E. J. Econ. Anal. Policy **10**, 53 (2010).

[4] M. N. Burton-Chellew, C. El Mouden, S. A. West, Conditional cooperation and confusion in public-goods experiments. Proc. Natl. Acad. Sci. U.S.A. **113**, 1291–1296 (2016). 26787890 10.1073/pnas.1509740113 PMC4747742

[5] T. R. Fosgaard, L. G. Hansen, E. Wengström, Framing and misperception in public good experiments. Scand. J. Econ. **119**, 435–456 (2017).

